# A Novel Peptide Enhances Therapeutic Efficacy of Liposomal Anti-Cancer Drugs in Mice Models of Human Lung Cancer

**DOI:** 10.1371/journal.pone.0004171

**Published:** 2009-01-12

**Authors:** De-Kuan Chang, Chin-Tarng Lin, Chien-Hsun Wu, Han-Chung Wu

**Affiliations:** 1 Institute of Cellular and Organismic Biology, Academia Sinica, Taipei, Taiwan; 2 Institute of Pathology, College of Medicine, National Taiwan University, Taipei, Taiwan; Dresden University of Technology, Germany

## Abstract

Lung cancer is the leading cause of cancer-related mortality worldwide. The lack of tumor specificity remains a major drawback for effective chemotherapies and results in dose-limiting toxicities. However, a ligand-mediated drug delivery system should be able to render chemotherapy more specific to tumor cells and less toxic to normal tissues. In this study, we isolated a novel peptide ligand from a phage-displayed peptide library that bound to non-small cell lung cancer (NSCLC) cell lines. The targeting phage bound to several NSCLC cell lines but not to normal cells. Both the targeting phage and the synthetic peptide recognized the surgical specimens of NSCLC with a positive rate of 75% (27 of 36 specimens). In severe combined immunodeficiency (SCID) mice bearing NSCLC xenografts, the targeting phage specifically bound to tumor masses. The tumor homing ability of the targeting phage was inhibited by the cognate synthetic peptide, but not by a control or a WTY-mutated peptide. When the targeting peptide was coupled to liposomes carrying doxorubicin or vinorelbine, the therapeutic index of the chemotherapeutic agents and the survival rates of mice with human lung cancer xenografts markedly increased. Furthermore, the targeting liposomes increased drug accumulation in tumor tissues by 5.7-fold compared with free drugs and enhanced cancer cell apoptosis resulting from a higher concentration of bioavailable doxorubicin. The current study suggests that this tumor-specific peptide may be used to create chemotherapies specifically targeting tumor cells in the treatment of NSCLC and to design targeted gene transfer vectors or it may be used one in the diagnosis of this malignancy.

## Introduction

Lung cancer is one of the most commonly diagnosed malignancies in developed countries and is a growing problem in developing countries [1]. There are two major types of lung cancer: non-small cell lung cancer (NSCLC) and small cell lung cancer (SCLC). NSCLC makes up approximately 80% of all lung cancer cases [2] and has a limited response rate to current chemotherapeutic agents, with tumor shrinkage in only 20% of patients and a two-year survival rate between 10% and 16% [3]. One major reason for this unsatisfactory outcome of chemotherapy is compromised drug delivery to the lung cancer tissues due to high interstitial fluid pressures (IFP) within the tumor [4]. Systemically administered chemotherapy cannot be adequately delivered into solid tumors because of the immature vasculature with abnormal architecture [5] and leaky, heterogeneous vessel walls [6] as well as the high IFP within tumor tissues [7], [8]. Furthermore, a lack of tumor specificity allows anti-cancer drugs to distribute indiscriminately to normal organs and tissues. Thus, cancer cells are exposed to a lower concentration of the drug than normal cells [9], resulting in not only decreased effectiveness but also increased toxicity. Therefore, it is important to develop a strategy to enhance the amount of drugs delivered to tumor tissues in a targeted way while sparing normal tissues.

Efforts are ongoing to improve the therapeutic index of anticancer agents, either by increasing the drug concentration inside the tumor or by decreasing it in normal host tissues [10]. Compared with conventionally administered chemotherapeutic agents, lipid- or polymer-based nano-medicine drug delivery systems have the advantage of improving the pharmacological and therapeutic properties of cytotoxic drugs [11]. Most small-molecule chemotherapeutic agents have a large volume of distribution on intravenous administration [12] and a narrow therapeutic window due to serious toxicity to normal tissues. By encapsulating drugs in nano-particles such as liposomes, scientists can significantly reduce the volume of distribution and increase the concentration of active drug within the tumor [13]. PEGylated liposomal doxorubicin (with brand names of Doxil in the US and Caelyx in Europe) [14] has been shown to significantly improve the therapeutic index of doxorubicin both in preclinical [15]–[17] and clinical studies [18]–[21]. Several drug delivery systems of this kind have been approved for marketing [22], [23].

Other than PEGylated liposomes, higher and more selective anti-cancer activity can be achieved through ligand-mediated targeting liposomes. In this novel drug delivery system, targeting moieties are coupled to the surface of liposomes to promote selective binding to tumor-specific antigens and facilitate the delivery of drug-containing liposomes to the intended cellular sites. This system has the advantages of a higher drug-to-carrier ratio than immunoconjugates and the multivalent presentation of ligands leading to increased binding avidity [24]. Researchers have already produced liposomes conjugated with various peptide ligands that specifically target certain tumor cells or tumor vasculature [25]–[29].

Because of the favorable selectivity and specificity, ligand-conjugated liposomal anticancer drugs are a promising approach for new chemotherapy research. The use of peptides as ligands to direct liposomes to tumors represents a potentially feasible method for increasing the specificity and effectiveness of liposome-containing drugs [26], [30]. Phage display is a technique of selecting targeting peptides, in which a peptide is expressed on the surface of a bacteriophage as a fusion-protein with one of the virion's own coat proteins [31]. Phage-displayed peptide libraries allows researchers to map protein-protein contacts such as B-cell epitopes [32]–[35] and receptor-ligand interactions [36]. Such peptide libraries can also be used to identify organ- and cell-type-specific peptides [26], [27], [37]–[39].

In this study, we used a phage-displayed peptide library to identify a novel peptide that bound specifically to NSCLC cell lines and surgical specimens from lung cancer patients. Liposomal doxorubicin and vinorelbine conjugated with this targeting peptide demonstrated enhanced accumulation of the drugs in tumor tissues and improved therapeutic index for human lung cancer xenografts in SCID mice.

## Results

### Isolation of phages binding to NSCLC cells

**Figure 1:**
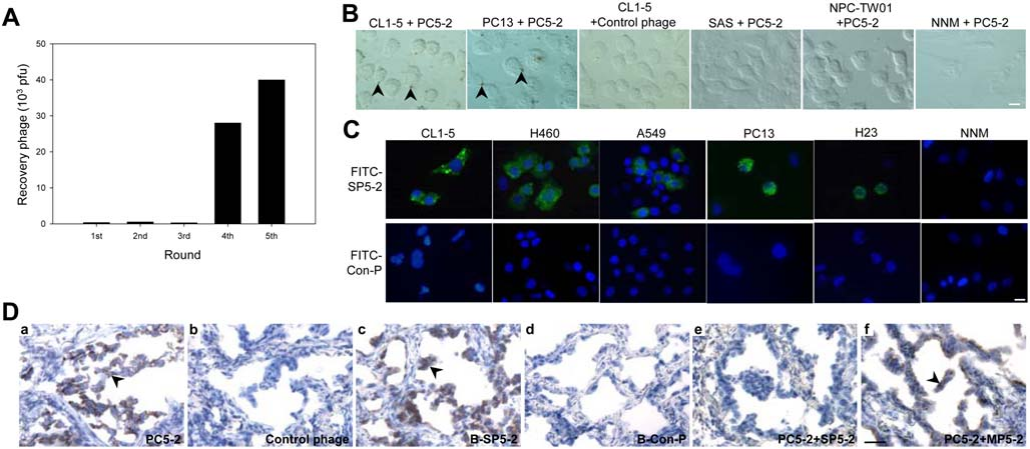
*In vitro* phage display screening for peptides that bind to NSCLC. (*A*) A phage-displayed random peptide library was used to select phages that bind to the NSCLC cell line CL1-5. (*B*) Visualization of PC5-2 binding to CL1-5 and PC13 lung cancer cells (arrowheads) with immunohistochemical staining. The control phage did not bind to CL1-5 cells. Scale bar: 10 µm. (*C*) The FITC-labeled peptide SP5-2 bound to five NSCLC cell lines but not to NPC-TW01 cells as detected by immunofluorescent staining. Scale bar: 10 µm. (*D*) Representative photomicrographs of tumor sections from surgical specimens of human lung cancer were detected using both PC5-2 (a, arrowhead) and biotinylated SP5-2 (c, arrowhead), respectively. In comparison, the control phage or biotinylated control peptide could not bind to these surgical specimens (b and d). PC5-2 was competitively inhibited by the synthetic peptide SP5-2 (e). Mutated peptide, MP5-2, lost this competition ability (f). Scale bar: 25 µm.

We used a phage-displayed random peptide library to isolate phages that were able to bind to NSCLC CL1-5 cells. After five rounds of affinity selection (biopanning) with CL1-5, cells increased the titer of phage by 40-fold (Fig. 1A). Enriched phages from the third to the fifth biopanning rounds were randomly selected. We then sequenced the phage clones with higher CL1-5-binding activities. Using the Genetics Computer Group (GCG) software analysis, we found that these selected phages (PC3-1, PC4-1, PC4-5, PC5-2 and PC5-4) displayed the consensus motif, tryptophan (W)-threonine (T)/tyrosine (Y)-tyrosine (Y) (Table 1). Interestingly, the phage PC5-2 appeared in the third (PC3-1), fourth (PC4-1) and fifth (PC5-2) biopanning rounds. During the biopanning rounds, the frequency of PC5-2 increased from 20% (1/5) in the third cycle to 90% (27/30) in the fifth cycle (Table 1). We chose to focus on the novel peptide displayed by PC5-2, TDSILRSYDWTY, for further study.

**Table 1:** Alignment of the phage-displayed peptide sequences selected by NSCLC cells.

Phage clone	Phage-displayed peptide sequence^a^	
5-2	TDSILRSYD**WTY**	27/30
5-4	DMPKQLLAP**WYY**	3/30
4-1	TDSILRSYD**WTY**	20/24
4-5	DMPKQLLAP**WYY**	2/24
4-9	SYPLSFLGPLIS	1/24
3-1	TDSILRSYD**WTY**	1/5
3-2	TQQPLEGHQLPY	1/5
3-3	TGVSWSVAQPSF	1/5
3-4	SVSVGMKPSPRP	1/5
3-5	SQWNSPPSSAAF	1/5

a Phage-displayed consensus amino acid sequences are shown in boldface.

### Identification of phage clones specifically binding to NSCLC cells

To investigate whether PC5-2 would bind to NSCLC cells, we used immunohistochemistry to locate the phage particles in different cell types. Our results showed that PC5-2 bound specifically to NSCLC cell lines including CL1-5 and PC13 (Fig. 1B, arrowheads). The control helper phage did not bind to CL1-5 cells. PC5-2 bound neither to other cancer cell lines, including oral cancer (SAS) and nasopharyngeal carcinoma cells (NPC-TW01), nor to normal epithelial cells (NNM) from nasal mucosa (Fig. 1B).

The CL1-5-binding ability of PC5-2 was further confirmed by a peptide competitive inhibition experiment using immunofluorescent staining. The results showed that the binding activity of the PC5-2 phage to CL1-5 cells was inhibited by the synthetic peptide SP5-2 in a dose-dependent manner. At a concentration of 27 µg/ml, SP5-2 completely inhibited the binding activity of PC5-2 (Fig. S1). The control phage did not bind to CL1-5 cells, and PC5-2 did not bind to NPC-TW01 in this assay (Fig. S1 and Text S2).

To further verify that the PC5-2 phage would bind to a target molecule expressed on the surface of CL1-5 cells, we measured PC5-2-bound cells using flow cytometry (Fig. S2 and Text S1, S2). A control phage was used to estimate non-specific background binding (Fig. S2b). The results showed that 42.6% of CL1-5 cells were bound by PC5-2 (Fig. S2c), and this binding was completely inhibited by 27 µg/ml of SP5-2 peptide (Fig. S2d). PC5-2 did not bind to SAS or NNM (Fig. S2 e and f).

### Binding of synthetic peptide SP5-2 with lung cancer cells and human lung cancer surgical specimens

To determine whether the peptide sequences displayed on PC5-2 would actually interact with NSCLC cells, we used fluorescein isothiocyanate (FITC)-labeled SP5-2 peptide (FITC-SP5-2) in place of the PC5-2 phage for a peptide-binding assay through immunofluorescent staining. FITC-SP5-2 specifically bound to all of the NSCLC cell lines we tested, including CL1-5, H460, A549, PC13 and H23, but did not bind to NNM. The same concentration of FITC-labeled control peptide (FITC-Con-P) revealed no such binding activity (Fig. 1C). We also evaluated the magnitude and specificity of SP5-2 binding using flow cytometry. The proportions of CL1-5, H460, A549, PC13 and H23 cells bound by SP5-2 were 43.0%, 45.8%, 44.3%, 20.1% and 44.0%, respectively (Fig. S3 and Text S2).

To determine whether this targeting ligand had an affinity for human lung cancer surgical specimens, we tested the reactivity of PC5-2 and SP5-2 with pulmonary adenocarcinoma cells using immunohistochemistry. Both PC5-2 and biotin-labeled SP5-2 (B-SP5-2) recognized the tumor cells of NSCLC surgical specimens (Fig. 1D a and c, arrowheads), and the control phage and biotin-labeled control peptide (B-Con-P) did not (Fig. 1D b and d). SP5-2 (TDSILRSYD**WTY**) competed with PC5-2 for binding to surgical specimens of pulmonary adenocarcinoma (Fig. 1De), but the same concentration of a mutated peptide, MP5-2 (TDSILRSYD**GGG**) did not (Fig. 1Df). Seventy-five percent (27/36) of the pulmonary adenocarcinoma specimens from 36 patients expressed a target molecule that was recognized by this peptide (Table S1). These data indicated that SP5-2 could recognize unidentified molecules expressed on NSCLC cell lines and actual cancer cells from the surgical specimens of lung cancer.

### Mice models for the study of PC5-2-targeting ability

**Figure 2:**
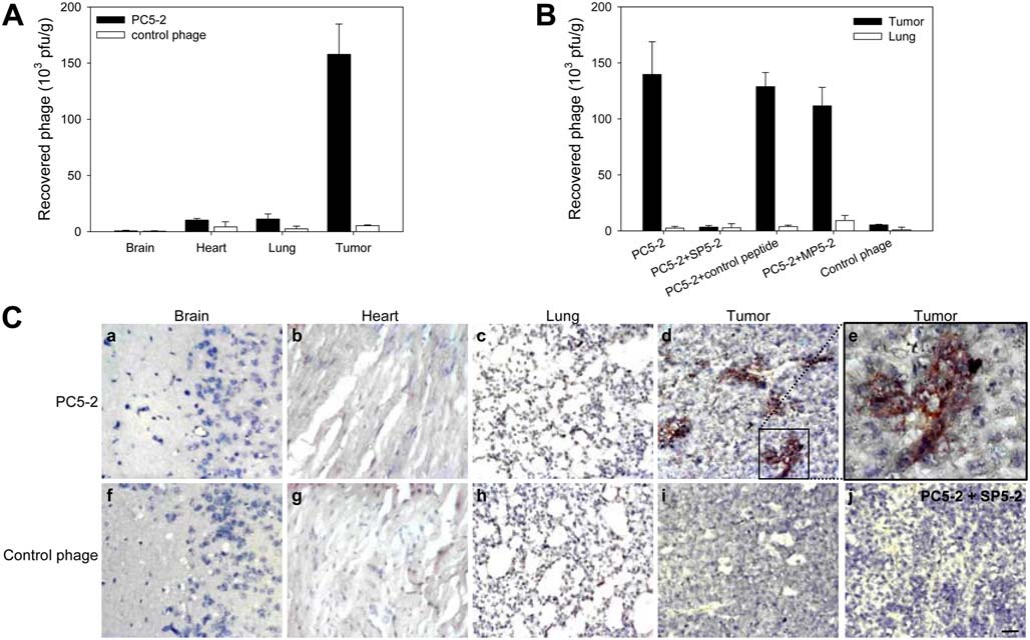
Verification of tumor-homing ability of PC5-2 *in vivo*. (*A*) SCID mice bearing human lung cancer xenografts received i.v. injections of PC5-2, and phage was recovered after perfusion. Recovery of PC5-2 from the tumor was markedly higher than from brain, heart or lungs. (*B*) Targeting activity of PC5-2 to tumor tissues was competitively inhibited by SP5-2 but not by control peptide or mutant peptide. (*C*) Immunohistochemical localization of PC5-2 after i.v. injection into mice with CL1-5-derived xenografts. The phage was localized on tumor tissues (d and e) and no localization was observed in normal organs such as the brain (a), heart (b), and lungs (c), or in the tissue sections treated with the control phage (f–i). The interaction of PC5-2 with the tumor section was inhibited by synthetic peptide SP5-2 (j). Scale bar: 25 µm.

To investigate the targeting ability of the PC5-2 phage *in vivo*, we injected phages into the tail vein of mice bearing CL1-5-derived tumors and then recovered them after perfusion. We determined the titers of the phage in tumor masses and normal control organs (brain, heart and lungs) [26], [30]. PC5-2 showed specific homing to tumor masses with concentrations 15-fold higher than its concentration in the control organs (Fig. 2A). Control helper phages did not show any specific targeting to tumor tissues (Fig. 2A).

The tumor-homing ability of PC5-2 was further confirmed by a peptide competitive inhibition experiment, in which synthetic peptide SP5-2, injected together with PC5-2, markedly inhibited the recovery of the phage from tumor masses (Fig. 2B). One hundred micrograms of SP5-2 inhibited 92% of PC5-2 binding to tumor masses, but the same concentration of a control peptide had no such inhibitory effect (Fig. 2B).

From *in vitro* phage display screening, we identified two clones (PC5-2 and PC5-4) with a consensus motif of W-T/Y-Y (Table 1). Like PC5-2, the tumor-homing ability of PC5-4 was also competitively inhibited by SP5-2 (Fig. S4 and Text S2), suggesting that these two phages may bind through this motif to the same target molecule on the plasma membrane of lung cancer cells. We proposed that these three amino acid residues might play a crucial role in homing to tumor tissues. To test this hypothesis, we changed these three amino acid residues in SP5-2 (TDSILRSYD**WTY**) to GGG in a mutant peptide, MP5-2 (TDSILRSYD**GGG**). Although the tumor-homing ability of PC5-2 had been markedly inhibited by the peptide SP5-2, this competitive inhibition was lost in MP5-2, which contains the GGG residue instead of the WTY residues in SP5-2 (Fig. 2B). These data indicate that the WTY residues were important for the binding ability of SP5-2 to NSCLC cells.

The tissue distribution of PC5-2 was also studied using immunostaining. We injected SCID mice bearing NSCLC xenografts with PC5-2 and then removed and fixed the tumor and control organs for localization of the phage particles. PC5-2 was found to localize in tumor tissues (Fig. 2Cd). At a higher magnification, the immunoreactivity of the phage was detected on the plasma membrane with some diffusion in the surrounding cytoplasm of tumor cells (Fig. 2Ce). There was no reaction product detected on normal organs such as brain, heart and lung tissues (Fig. 2C a–c), nor on tumor tissues treated by the control phage (Fig. 2Ci). The specific targeting of PC5-2 to the NSCLC xenograft was inhibited by the synthetic peptide SP5-2 in the *in vivo* homing experiment (Fig. 2Cj).

### Therapeutic efficacy of SP5-2-mediated targeting liposomes

To determine whether the lung cancer-targeting peptide SP5-2 could be used to improve the chemotherapeutic efficacy of cancer treatment, we coupled the peptide to liposomes containing anti-cancer drugs. SCID mice bearing size-matched, CL1-5-derived xenografts were treated with (1) SP5-2-conjugated liposomal doxorubicin (SP5-2-LD), (2) mutant peptide-conjugated liposomal doxorubicin (MP5-2-LD), (3) non-targeted liposomal doxorubicin (LD), (4) free doxorubicin (FD), or (5) equivalent volumes of phosphate-buffered saline (PBS). All the formulations were injected intravenously (i.v.) at a total doxorubicin dosage of 8 mg/kg (1 mg/kg twice a week for a total of eight injections).

**Figure 3:**
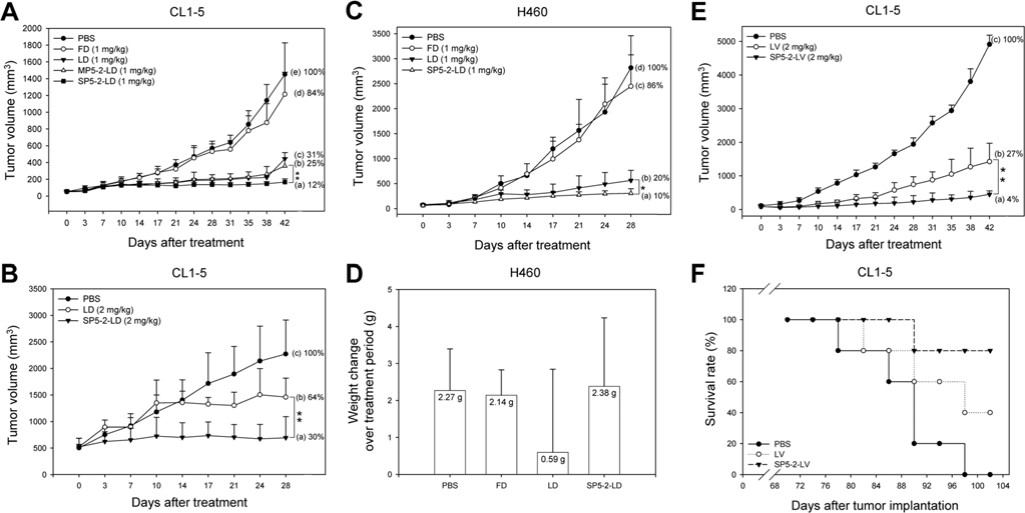
Treatment of SCID mice bearing human lung cancer xenografts with SP5-2-LD or SP5-2-LV. (*A*) Median tumor volume over time in mice treated with PBS, FD, LD, MP5-2-LD, or SP5-2-LD. Tumor growth was markedly suppressed in the SP5-2-LD treated group. SP5-2-LD has higher therapeutic efficacy than LD and MP5-2-LD (*n* = 6 in each group; **P<0.01). (*B*) Mice bearing size-matched CL1-5-derived lung cancer with tumor size of approximately 500 mm^3^ were treated with SP5-2-LD, LD, or PBS (*n* = 6 in each group, **P<0.01). (*C*) Mice bearing H460-derived lung cancer were treated with SP5-2-LD, LD, FD, or PBS (*n* = 6 in each group, *P<0.05). (*D*) The effects of different treatments on change in body weight over the treatment period (*n* = 6 in each group). (*E*) Mice bearing CL1-5-derived lung cancer were treated with SP5-2-LV, LV, or PBS (*n* = 6 in each group; **P<0.01). (*F*) A Kaplan-Meier survival curve showed longer lifespan of mice treated with SP5-2-LV than those treated with LV and PBS (n = 5 in each group).

The tumors in mice that received SP5-2-LD (Fig. 3A group a) were significantly smaller than those in the MP5-2-LD, LD, FD, and PBS groups (*P*<0.01) (Fig. 3A group b–e). The tumor sizes in the LD and MP5-2-LD groups were 2.1 and 2.6 times larger than those in the SP5-2-LD group, respectively. The tumor sizes in the FD and control PBS groups were 7.0 and 8.3 times larger than that in the SP5-2-LD group, respectively (Fig. 3A). Interestingly, the greater therapeutic efficacy of SP5-2-LD was lost when the WTY motif in the peptide had been changed to GGG in MP5-2-LD. Free doxorubicin exhibited little therapeutic efficacy at this concentration, as the tumor size in this group was only 16% smaller than that in the PBS group (Fig. 3A).

To verify whether large xenografts would respond to SP5-2-LD treatment, mice bearing large lung cancer xenografts (500 mm^3^) were assigned to three treatment groups. After a course of doxorubicin treatment with a total dosage of 16 mg/kg (2 mg/kg twice a week for eight injections), the tumor sizes in the control PBS and LD groups gradually increased to 3.3 and 2.1 times the tumor size in the SP5-2-LD group (*P*<0.01) (Fig. 3B). These results revealed that SP5-2-LD also increased the therapeutic efficacy of doxorubicin in SCID mice bearing large lung cancer xenografts.

To further test whether SP5-2 would optimize the therapeutic index in lung cancer treatment, SP5-2-LD was used to treat a different type of lung cancer xenograft (H460-derived tumor). SCID mice bearing size-matched, H460-derived xenografts were treated with SP5-2-LD, LD, FD, or equivalent volumes of PBS, through i.v. injection at a total doxorubicin dosage of 8 mg/kg (1 mg/kg twice a week for eight injections). The final tumor size in the SP5-2-LD group was significantly smaller than those in the LD, FD and PBS groups (*P*<0.05). Mice in the LD, FD and PBS groups had tumors with sizes 2.0-, 8.6- and 10.0-fold larger than that in the SP5-52-LD group (Fig. 3C). Free doxorubicin at this concentration reduced the tumor size by only 14% compared with PBS groups. Not only was tumor growth markedly suppressed in the SP5-2-LD group (Fig. 3C), the body weight of the mice in this group increased by 10.3% (2.38 g) at the end of the treatment period. In contrast the LD-treated mice had a smaller increase in body weight of 2.4% (0.59 g) (Fig. 3D).

To further confirm SP5-2 could increase the therapeutic index for lung cancer, we linked the SP5-2 peptide to another anti-cancer drug, liposomal vinorelbine (SP5-2-LV) and tested its efficacy against lung cancer xenografts. SCID mice bearing size-matched CL1-5-derived xenografts were given i.v. injections of SP5-2-LV, liposomal vinorelbine (LV), or equivalent volumes of PBS at a total vinorelbine dose of 24 mg/kg (2 mg/kg twice a week for twelve injections). The tumor-bearing mice treated with SP5-2-LV (Fig. 3E, group a) had significantly smaller tumors than the LV and PBS groups (*P*<0.01) (Fig. 3E, group b and c). The tumor size in the LV group was 6.75-fold larger than the SP5-2-LV group. The average tumor size in the control PBS group was 25-fold larger than the SP5-2-LV group (Fig. 3E). To assess side effects of the treatments, the mice were weighed twice a week. The body weight of mice increased by 5.7% (1.37 g) in the SP5-2-LV group and by 2.4% (0.58 g) in the LV group at the end of the treatment period (data not shown).

Finally, we compared the survival rates of tumor-bearing mice after treatment with SP5-2-LV, LV, or PBS over 102 days. All five animals in the PBS-treated group died (survival rate 0%). Three mice died in the LV-treated group (survival rate 40%). In the SP5-2-LV-treated group, however, the survival rate was 80%, significantly higher than the other two groups (Fig. 3F). These experiments demonstrate that SP5-2 increased the therapeutic efficiency of liposome-encapsulated doxorubicin and vinorelbine with less toxicity.

### Tumor localization and biodistribution of SP5-2-conjugated targeting liposomes

**Figure 4:**
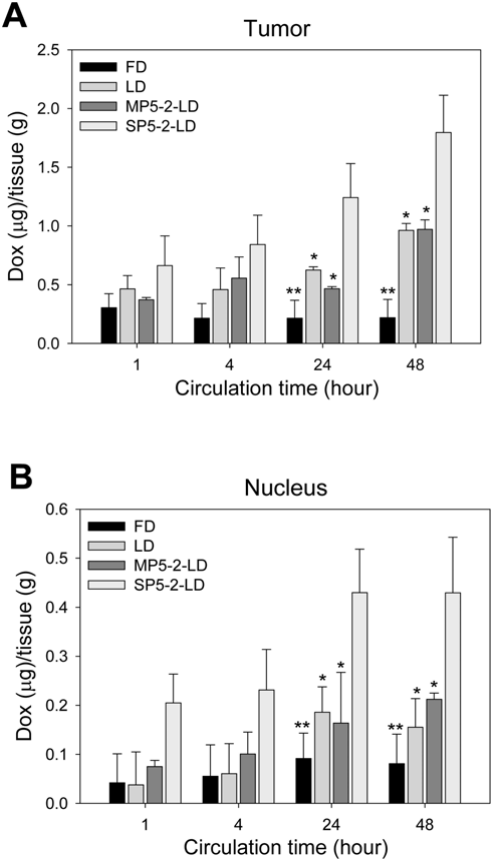
Tumor localization of different formulations of liposomal and free doxorubicin in a NSCLC xenograft mouse model. At selected time points (1, 4, 24 and 48 hours) after injection, doxorubicin concentration in tumor tissues (*A*) and nuclei of cancer cells (*B*) was measured. Doxorubicin concentration inside tumor tissues and nuclei in the SP5-2-LD group was higher than in the FD, LD, and MP5-2-LD groups (n = 3 at each time point, *P<0.05, **P<0.01).

The biodistribution and tumor localization of SP5-2-LD, MP5-2-LD, LD, and FD were estimated by measuring the intrinsic auto-fluorescence signal of doxorubicin in mice with NSCLC xenografts. Doxorubicin (*M*_r_ 543.54), a small-molecule chemotherapeutic agent as its *M*_r_ is <1000, has a poor pharmacokinetic profile, and its blood concentration drops to background level within one hour after administration (Fig. S5A and Text S2). The pharmacokinetic profile of various liposomal doxorubicin formulations, including SP5-2-LD, MP5-2-LD, and LD, were markedly greater than FD (Fig. S5A). The area under the concentration-time curve (AUC_0–48 hours_) of doxorubicin in tumor tissues was 10.4 µg·hr/g, 31.5 µg·hr/g, 29.0 µg·hr/g, and 59.9 µg·hr/g in the FD, LD, MP5-2-LD, and SP5-2-LD groups, respectively (Table S2). The mean intra-tumor doxorubicin concentration in the SP5-2-LD-treated group was 5.7-, 1.9- and 2.1-fold higher than the intra-tumor doxorubicin concentration in the FD, LD, and MP5-2-LD groups (Fig. 4A and Table S2).

To assess the bioavailability of the liposomal drugs, we used nuclear doxorubicin accumulation as an indicator of drug cytotoxicity [40]. The AUC_0–48 hours_ of bioavailable doxorubicin (i.e., bound to nuclei) for FD, LD, MP5-2-LD, and SP5-2-LD was 3.7 µg·hr/g, 6.7 µg·hr/g, 7.5 µg·hr/g, and 17.7 µg·hr/g, respectively (Table S2). The intra-tumor nuclear doxorubicin concentration in the SP5-2-LD group was 4.8-, 2.6- and 2.4-fold higher than the nuclear doxorubicin concentration in the FD, LD, and MP5-2-LD groups (Fig. 4B and Table S2). Doxorubicin concentration inside tumor tissues and nucleus was not significantly different between the LD and MP5-2-LD groups (Fig. 4 and Table S2).

### Enhanced tumor drug delivery and therapeutic efficacy of SP5-2-conjugated liposomes

**Figure 5:**
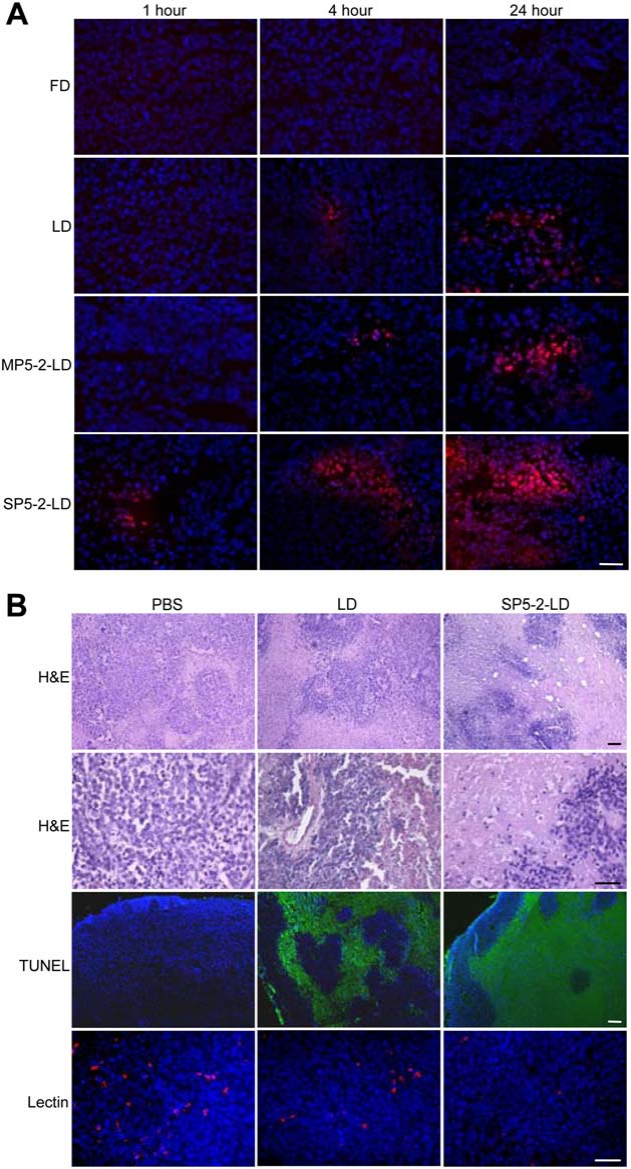
SP5-2 enhanced drug delivery to tumor tissues and increased therapeutic efficacy. (*A*) Representative two-color images showing the distribution of doxorubicin (red) in relation to nuclei (blue) in tissue sections. Accumulation of doxorubicin in tumor tissues was shown at 1, 4 and 24 hours post-injection. Bar, 50 µm. (*B*) Histopathology and fluorescent staining of tumor tissues in each treatment group examined after staining with H&E, TUNEL (green), lectin (red), and DAPI (blue). Bar, 50 µm. Enhancement of drug accumulation in tumor tissues correlated with the increased therapeutic efficacy.

To compare the drug delivery profile of the four doxorubicin formulations, we tried to detect the drug in tumor tissues using the fluorescence microscope. Images from all tumors showed that doxorubicin was visualized in the tumor nuclei one hour after SP5-2-LD was administered, but not after other formulations were injected (Fig. 5A). Over time, areas of tumor sections with detectable doxorubicin increased. The areas with detectable doxorubicin were significantly larger in SP5-2-LD-treated tumors than that in FD, LD, and MP5-2-LD-treated tumors at each time point (Fig. 5A). The MP5-2-LD formulation had the same distribution pattern as LD, but the regions of FD-treated tumors showed no detectable doxorubicin (Fig. 5A).

When the tumor tissues in each treatment group (Fig. 3C) were examined by H&E staining, markedly disseminated necrotic/apoptotic areas were present throughout the sections of SP5-2-LD-treated xenografts (Fig. 5B). In addition, terminal deoxynucleotidyl transferase-mediated dUTP nick end labeling (TUNEL) was used to identify apoptotic cells and tomato lectin was applied to detect tumor blood vessels. The tumors had larger apoptotic areas and a lower density of tumor blood vessels in the SP5-2-LD-treated group than in the LD- and PBS-treated groups (Figs. 5B and S6).

The greater accumulation of anti-cancer drugs in tumor tissues and more bioavailable doxorubicin in cancer cell nuclei from the ligand-conjugated liposomes further demonstrated that the SP5-2 peptide recognized the target molecule on the surface of lung cancer cells and thus increased direct drug delivery to the tumor.

## Discussion

The therapeutic efficacy of anticancer drugs can be achieved by enhancing the drug formulation with molecules that preferentially bind to tumor tissues [22], [26], [37], [41]. Phage display biopanning on intact cell allows for the isolation of highly specific peptides that target tumor-associated antigens. Anti-cancer regimens armed with these peptides can be used as “cruise missiles” that are precisely guided to the cancer cells and deliver high enough doses to kill these cells with minimal damage to normal tissues.

In this study, we identified a NSCLC-targeting peptide and demonstrated its improved therapeutic efficiency in animal models. Specifically, we found a phage clone PC5-2 that made up 90% of the selected phages binding to CL1-5 cells after five rounds of biopanning (Table 1). Immunohistochemistry and flow cytometry assays confirmed that PC5-2 bound to NSCLC cell surface. The same binding results were observed in the cognate synthetic peptide SP5-2, which replaced PC5-2 (Fig. 1 and Figs. S1, S2, S3). These experiments supported that this targeting peptide can specifically bind to the cell surface of NSCLC cell lines. *In vivo* experiments also consolidated the homing ability of the peptide. The expression of the SP5-2 peptide (TDSILRSYDWTY) guides the phage to accumulate in NSCLC xenografts but not in normal organs (Fig. 2). The binding activity of PC5-2 to tumor tissues was inhibited by the synthetic peptide SP5-2 (Fig. 2B), indicating that PC5-2 interacted with NSCLC cells by its displayed peptide and not by another part of the phage particle. That the mutated synthetic peptide, MP5-2, did not inhibit the PC5-2 binding demonstrated the importance of the WTY motif in the binding activity (Fig. 2B). Moreover, this phenomenon was observed in immunohistochemical staining of surgical specimens from human lung cancer (Fig. 1D e and f), in animal models for ligand-targeted chemotherapy (Fig. 3A) and in tumor localization of doxorubicin delivered by SP5-2-conjugated targeting liposomes (Figs. 4 and 5A).

Immunohistochemistry assessments on pulmonary adenocarcinoma surgical specimens demonstrated that SP5-2 has a clinical potential as imaging probes to identify NSCLC or drug delivery agents for the treatment of NSCLC. Both PC5-2 and biotin-labeled SP5-2 detected pulmonary adenocarcinoma surgical specimens in our experiments (Fig. 1D). Seventy-five percent (27/36) of pulmonary adenocarcinoma specimens from 36 patients expressed a target molecule that was recognized by the peptide (Table S1). SP5-2, but not MP5-2, competed with PC5-2 for binding to pulmonary adenocarcinoma surgical specimens (Fig. 1D), which further confirmed the specificity of this targeting ligand.

In previous studies, doxorubicin has largely been used in cancer treatment because of its broad spectrum of antitumor activity. However, the efficacy of doxorubicin in the treatment of NSCLC remains unsatisfactory with a response rate of 15% [42]. This is in part a result of suboptimal doses within the tumor due to indiscriminate drug distribution throughout the body and severe toxicity to normal tissues and organs. Liposomal encapsulation with a targeting ligand may be an effective strategy to deliver the drug directly to tumor cells. Our data revealed that this peptide markedly increased the therapeutic efficacy of liposomal chemotherapies and resulted in higher survival rates in mice with human lung cancer xenografts, and produced limited side effects on the animals (Fig. 3). This peptide increased the therapeutic index of not only doxorubicin but also of vinorelbine, a vinca alkaloid used to treat advanced NSCLC [3]. Furthermore, we observed decreased vessel density and substantially increased cell apoptosis in tumor tissues after the targeting liposome treatment (Figs. 5B and S6). This ligand-mediated liposomal formulation is potentially superior to conventional anti-cancer therapy for NSCLC.

Our *in vivo* pharmacokinetic studies in mice showed that liposomal doxorubicin dramatically changed the transportation and distribution of doxorubicin in the heart, lungs, kidneys, and liver in mice (Figs. 4 and S5). These results echo other similar investigations [43], [44]. With prolonged presence of liposomes in circulation, more doxorubicin was taken up by tumor cells than conventional doxorubicin administration (Fig. 4); this finding was further confirmed in fluorescence signaling of doxorubicin in tumor tissues (Fig. 5A). This passive targeting phenomenon of non-ligand conjugated liposomes is called the “enhanced permeability and retention effect” [45], [46]. In our study, the doxorubicin concentration in the LD-treated group was three times that of the FD group (Table S2). However, the tumors in the SP5-2-LD group had even higher doxorubicin concentration that was 1.9- and 2.1-fold higher than those in the LD and MP5-2-LD groups (Table S2). The bioavailable drug in the nuclei of cancer cells in the SP5-2-LD was also 2.6- and 2.4-fold higher than those in the LD and MP5-2-LD groups (Table S2). These results indicated that this peptide directly delivered the chemotherapeutic drug to intended targets. Enhancement of drug accumulation in tumor tissues correlated with the increased therapeutic efficacy (Figs. 3, 4 and 5). The peptide-functionalized liposomes were found to have important clinical potential in a targeted drug delivery system. In order to for them to be used clinically, however, a final targeting liposome construct will need to be selected after each component, i.e. peptide ligands, conjugation methodologies, and liposomal drugs, have been optimized.

In conclusion, using phage display peptide libraries to screen for peptides that bind to NSCLC cells, we identified several novel peptides including SP5-2 that specifically bound to the cell surface of NSCLC cells both *in vitro* and *in vivo*. Linking SP5-2 to liposomes containing doxorubicin and vinorelbine increased the therapeutic efficacy and survival rates in mice with human NSCLC xenografts because of enhanced tumor apoptosis and decreased tumor angiogenesis. Quantitation and visualization of doxorubicin levels also showed increased drug concentration in tumor tissues in this formulation, highlighting the enhancement in both the delivery and penetration of doxorubicin into the tumor. Our results indicate that the SP5-2 tumor targeting peptide may be used as imaging probes of NSCLC and targeting ligands for liposomal delivery systems to increase the efficacy of chemotherapy for NSCLC.

## Materials and Methods

### Cell lines and cultures

Lung cancer cell lines (A549, CL1-5, H23, H460, and PC13), a nasopharyngeal carcinoma cell line (NPC-TW01), an oral cancer cell line (SAS), and human normal nasal mucosal epithelial (NNM) cells were used in this study. The NNM cells were a primary culture derived from a nasal polyp [27]. The A549, H23, and H460 lines were obtained from the American Type Culture Collection. The CL1-5 line was established by Chu et al. [47]. The A549, CL1-5, H23 and H460 cells were grown in RPMI 1640 (Gibco, CA, USA) containing 10% fetal bovine serum (FBS, Gibco, CA, USA) at 37°C in a 5% CO_2_ incubator. The NPC-TW01, PC13, SAS, and NNM cells were grown in DMEM (Gibco, CA, USA) containing 10% FBS at 37°C in a 10% CO_2_ incubator.

### Phage display biopanning procedures

CL1-5 cells were first incubated with UV-treated inactive control helper phage (insertless phage). The phage-displayed peptide library (New England BioLabs, MA, USA), which initially contained 5×10^10^ plaque-forming units (pfu), was then added. The bound phages were eluted with a lysis buffer (150 mM NaCl, 50 mM Tris-HCl, 1 mM EDTA, 1% NP-40, 0.5% sodium deoxycholate, 0.1% SDS, pH 7.4) on ice. This eluted phage pool was amplified and titered in an *Escherichia coli* ER2738 culture (New England BioLabs, MA, USA). Recovered phages were used as input for the next round of panning as described previously [26], [27].

### Identification of phage clones using cellular enzyme-linked immunosorbent assay (ELISA)

Ninty-six-well ELISA plates (Falcon, CA, USA) were seeded with either cancer or control cells. Individual phage particles were added to the cell-coated plates and incubated, followed by incubation with horseradish peroxidase (HRP)-conjugated mouse anti-M13 monoclonal antibody (mAb) (Pharmacia, Uppsala, Sweden) and subsequently with the peroxidase substrate *o*-phenylenediamine dihydrochloride (Sigma, MO, USA). The reaction was stopped and absorbance was measured at 490 nm using an ELISA reader. The selected phage clones were further analyzed using DNA sequencing. The sequencing was performed with the primer 5′-CCCTCATAGTTAGCGTAACG-3′ corresponding to the pIII gene sequence. The phage-displayed peptide sequences were translated and aligned using GCG program.

### Peptide synthesis and labeling

The synthetic targeting peptide SP5-2 (TDSILRSYDWTY), mutant peptide MP5-2 (TDSILRSYDGGG), and control peptide (RLLDTNRPLLPY) [27] were synthesized (Invitrogene, Inc., CA, USA) and purified using reverse-phase high-performance liquid chromatography to >95% purity. Conjugation of these peptides with FITC or biotin was performed through the addition of FITC or biotin to the peptide amino terminus by the same company.

### Identification of phages and synthetic peptides binding to cancer cells

Cells were plated and grown to about 80% confluence on cover slips. The cover slips were treated with 3% hydrogen peroxide plus 0.1% NaN_3_ to block endogenous peroxidase activity, and then incubated with phages. After the cover slips had been washed and fixed with 3% paraformaldehyde, they were incubated with HRP-labeled mouse anti-M13 mAb and treated with peroxidase substrate. For the peptide binding assays, 30 µg/ml FITC-labeled SP5-2 or control peptide was added on each cover slip and incubated. They were counterstained with Hoechst 33258 (Molecular Probes, OR, USA) and mounted with a mounting solution (Vector, CA, USA). The cells were then examined under a Leica universal microscope. The images were merged using the SimplePCI software (C-IMAGING, PA, USA).

### Binding of phages and synthetic peptides to surgical specimens of lung cancer

For localization of peptide binding on lung cancer tissues, frozen sections of NSCLC tissues were prepared and incubated with phage clones or biotin-labeled peptides. For the peptide competitive inhibition assay, phages were mixed with the synthetic targeting or mutant peptide. The slides were subjected to routine immunohistochemical staining [26], [30]. All surgical specimens were obtained from the tissue bank of National Taiwan University Hospital (NTUH) with approval from the Institutional Review Board in NTUH (IRB9461702021).

### *In vivo* homing experiments and tissue distribution of phages

SCID mice were injected subcutaneously (s.c.) in the dorsolateral flank with 1×10^7^ human NSCLC cells. The mice bearing size-matched lung cancer xenografts (approximately 500 mm^3^) were injected i.v. with 10^9^ pfu of the targeting or control phage. After perfusion, xenograft tumors and mouse organs were removed and homogenized. The phages bound to each tissue sample were recovered through the addition of ER2738 bacteria and titered on IPTG/X-Gal agar plates. In the peptide competitive inhibition experiments, the phages were injected along with 100 µg synthetic targeting peptide, control peptide or mutant peptide. The organs and tumor masses were fixed in Bouin's solution (Sigma, MO, USA). After fixation, the samples were embedded in paraffin blocks. The paraffin sections were deparaffinized, rehydrated, and subjected to immunostaining using the mouse M13 mAb as described above.

### Preparation of synthetic peptide-conjugated liposomes containing doxorubicin or vinorelbine

Peptide-conjugated liposomes containing doxorubicin or vinorelbine were prepared as described in other studies [26], [27]. Briefly, the peptide was coupled to NHS-PEG-DSPE [N-hydroxysuccinimido-carboxyl-polyethylene glycol (MW, 3400)-derived distearoylphosphatidyl ethanolamine] (NOF Corporation, Japan) in a 1∶1.5 molar ratio. The reaction was completed and confirmed by quantitation of the remaining amino groups using TNBS (Trinitrobenzenesulfonate) reagent (Sigma, MO, USA). Doxorubicin and vinorelbine were encapsulated in liposomes through a remote loading method at a concentration of 1 mg of drug per 10 µmol phospholipids. Peptidyl-PEG-DSPE was transferred to pre-formed liposomes after co-incubation at a transition temperature of the lipid bilayer. There were 500 peptide molecules per liposome as described previously [48].

### Pharmacokinetic and biodistribution studies

SCID mice bearing NSCLC xenografts (∼500 mm^3^), were injected in the tail vein with various formulations of liposomal doxorubicin (SP5-2-LD, MP5-2-LD, and LD) and free doxorubicin at a dose of 2 mg/kg. At selected time points, three mice in each group were anaesthetized and sacrificed. Blood samples were collected through submaxillary punctures, and plasma samples were prepared. After perfusion, xenograft tumors and mouse organs were removed and homogenized. Procedures for isolating tumor cell nuclei and extracting nuclear doxorubicin were carried out according to previous reports [40], [49]. Total doxorubicin concentration was measured using a method described by Mayer et al. [49]. Total doxorubicin was quantified using spectrofluorometry at *λ*_ex_ 485/20 nm and *λ*_em_ 645/40 nm (Synergy HT Multi-Detection Microplate Reader, BioTek Instruments, Winooski, VT 05404 USA).

To correct for background fluorescence, a standard curve was obtained by spiking tissue extracts derived from mice that did not receive doxorubicin. Tissue concentrations of doxorubicin were expressed as microequivalents per milliliter of plasma or per gram of tissue. A standard doxorubicin curve was prepared in control homogenates following the same extraction procedure as above. Drug levels were estimated on the basis of doxorubicin fluorescent equivalents.

To determine the presence of the drug localized in tumor tissues, doxorubicin autofluorescence was detected using a Zeiss Axiovert 200 M inverted microscope with a 100 W HBO mercury light source equipped with a 546/12 nm excitation and a 590 nm emission filter set. Tissue sections were imaged with a FLUAR 10×/0.50 NA lens and captured with a Roper Scientific CoolSnap HQ CCD camera. All images were captured in 8-bit signal depth and subsequently pseudo-colored.

### Animal model for the study of ligand-targeted therapy

Mice 4–6 weeks of age were injected s.c. in the dorsolateral flank with human NSCLC cells. Mice with size-matched tumors (approximately 50–100 mm^3^) were then randomly assigned to different treatment groups and injected with SP5-2-LD or SP5-2-LV, or LD or LV through the tail vein. The dosage of SP-5-2-LD was 1 mg/kg injected twice a week for four weeks, and that of SP5-2-LV was 2 mg/kg injected twice a week for six weeks. Mice bearing large tumors (approximately 500 mm^3^) were treated with SP5-2-LD for eight times (2 mg/kg, twice a week for four weeks). Mouse body weights and tumor sizes were measured twice a week. Tumor volumes were calculated using the equation: length×(width)^2^×0.52. Animal care was carried out in accordance with guidelines of Academia Sinica, Taiwan.

### Terminal deoxynucleotidyl transferase–mediated dUTP nick end labeling (TUNEL) staining

The frozen tumor tissue sections were incubated with terminal deoxynucleotidyl transferase-mediated dUTP nick end labeling reaction mixture (Roche Diagnostics) at 37°C for an hour. The slides were counterstained with mounting medium with DAPI (Vector Laboratories). The slides were then visualized under a fluorescent microscope.

### Vessel staining

Tissues were removed from treated mice, fixed with 4% paraformaldehyde and embedded with paraffin. Blood vessels were detected by staining of *Lycopersicon esculentum* (tomato) lectin conjugated to biotin (Vector, CA, USA). The biotinylated lectin was visualized with streptavidin-conjugated rhodamine (Pierce, IL, USA).

### Statistical analyses

We analyzed the data of phage titer, tumor volume, body weight, and doxorubicin concentration using two-sided unpaired Student's *t*-test. We considered a *P* value below 0.05 as significant for all analyses. All values are represented as mean±standard deviation.

## Supporting Information

Text S1 Supporting Information and figure legends
(0.02 MB DOC)

Text S2 Supplementary figure legend
(0.03 MB DOC)

Figure S1 Identification of PC5-2 binding to NSCLC cells. Representative microscopy images of CL1-5 cells stained with propidium iodide (a–f, red), anti-M13 monoclonal antibody (g–l, green), and merge (m–r). The binding of PC5-2 to CL1-5 cells (a, g, m) was inhibited by 3 µg/ml (b, h, n), 9 µg/ml (c, i, o), and 27 µg/ml (d, j, p) of SP5-2 in a dose-dependent manner. The control phage and PC5-2 did not bind to CL1-5 cells (e, k, q) and NPC-TW01 cells, respectively. Scale bar: 10 µm.
(3.54 MB TIF)

Figure S2 The binding activity of PC5-2 to NSCLC cells was analyzed by flow cytometry. The samples included cells only (a, green), control phage (b, red), and PC5-2 (blue). The percentages indicate the fraction of cells gated for positive binding (c). The binding of PC5-2 to CL1-5 cells was completely inhibited by 27 µg/ml of SP5-2 (d). PC5-2 did not bind to SAS and NNM cells (e, f).
(2.42 MB TIF)

Figure S3 FITC-labeled SP5-2 binding to NSCLC cells was analyzed by flow cytometry. The red line superimposed on all panels represents the control phage. CL1-5, H460, A549, PC13, and H23 panels showed positive binding, and the percentages were 43, 45.8, 44.3, 20.1, and 44, respectively. There was only background level in NPC-TW01 cells.
(3.04 MB TIF)

Figure S4 Tumor homing ability of PC5-4 phage. SCID mice bearing NSCLC xenografts were injected i.v. with PC5-4, and phage was recovered after perfusion. Recovery of PC5-4 from the tumor was higher than from control organs. Targeting activity of PC5-4 to tumor tissues was inhibited by SP5-2.
(0.18 MB TIF)

Figure S5 Biodistribution of different formulations of liposomal and free doxorubicin in a NSCLC xenograft mouse model. Mice were i.v. injected with SP5-2-LD, MP5-2-LD, LD, and FD in a single dose of 2 mg/kg. At selected time points (1, 4, 24 and 48 hours) after injection, doxorubicin concentration in blood, and organs were measured (n = 3 at each time point).
(0.62 MB TIF)

Figure S6 Changes of lectin-reactive vessels in SP5-2-LD-treated tumors. Positive lectin localization was quantified by pixel area count (Metamorph software, MDS, Inc.) under low power magnification.
(0.20 MB TIF)

Table S1 Detection of human lung cancer surgical specimens by PC5-2 using immunohistochemistry
(0.06 MB DOC)

TableS2 Tumor pharmacokinetics of free doxorubicin versus liposomal doxorubicin formulations
(0.03 MB DOC)
