# Supplementary material for: A Novel Peptide Enhances Therapeutic Efficacy of Liposomal Anti-Cancer Drugs in Mice Models of Human Lung Cancer
Source: PLoS One. 2009 Jan 12;4(1):e4171. doi: 10.1371/journal.pone.0004171 (PMC2614347; doi:10.1371/journal.pone.0004171)
Supplement: Text S1 — Supporting Information and figure legends (0.02 MB DOC) [file pone.0004171.s001.doc]

**Supporting Information**

**SI Text**

**Flow cytometry**

The lung and other cancer cell lines were collected using PBS containing 50 mM EDTA, then incubated with phages or FITC-labeled peptides. For the peptide competitive inhibition assay, phages were mixed with different concentrations of the synthetic peptide and the mixture was incubated with the cells. After washing, the phage-bound cells were incubated with anti-M13 mAbs and then treated with FITC-conjugated goat anti-mouse IgG antibody. Cells were washed and analyzed with a flow cytometer (Becton Dickinson)
