# Supplementary material for: A Novel Peptide Enhances Therapeutic Efficacy of Liposomal Anti-Cancer Drugs in Mice Models of Human Lung Cancer
Source: PLoS One. 2009 Jan 12;4(1):e4171. doi: 10.1371/journal.pone.0004171 (PMC2614347; doi:10.1371/journal.pone.0004171)
Supplement: Text S2 — Supplementary figure legend (0.03 MB DOC) [file pone.0004171.s002.doc]

**SI Figure Legends**

**Fig. S1.** Identification of PC5-2 binding to NSCLC cells. Representative microscopy images of CL1-5 cells stained with propidium iodide (a-f, red), anti-M13 monoclonal antibody (g-l, green), and merge (m-r). The binding of PC5-2 to CL1-5 cells (a, g, m) was inhibited by 3 µg/ml (b, h, n), 9 µg/ml (c, i, o), and 27 µg/ml (d, j, p) of SP5-2 in a dose-dependent manner. The control phage and PC5-2 did not bind to CL1-5 cells (e, k, q) and NPC-TW01 cells, respectively. Scale bar: 10 µm.

**Fig. S2.** The binding activity of PC5-2 to NSCLC cells was analyzed by flow cytometry. The samples included cells only (a, green), control phage (b, red), and PC5-2 (blue). The percentages indicate the fraction of cells gated for positive binding (c). The binding of PC5-2 to CL1-5 cells was completely inhibited by 27 µg/ml of SP5-2 (d). PC5-2 did not bind to SAS and NNM cells (e, f).

**Fig. S3.** FITC-labeled SP5-2 binding to NSCLC cells was analyzed by flow cytometry. The red line superimposed on all panels represents the control phage. CL1-5, H460, A549, PC13, and H23 panels showed positive binding, and the percentages were 43, 45.8, 44.3, 20.1, and 44, respectively. There was only background level in NPC-TW01 cells.

**Fig. S4.** Tumor homing ability of PC5-4 phage. SCID mice bearing NSCLC xenografts were injected i.v. with PC5-4, and phage was recovered after perfusion. Recovery of PC5-4 from the tumor was higher than from control organs. Targeting activity of PC5-4 to tumor tissues was inhibited by SP5-2.

**Fig. S5.** Biodistribution of different formulations of liposomal and free doxorubicin in a NSCLC xenograft mouse model. Mice were i.v. injected with SP5-2-LD, MP5-2-LD, LD, and FD in a single dose of 2 mg/kg. At selected time points (1, 4, 24 and 48 hours) after injection, doxorubicin concentration in blood, and organs were measured (n=3 at each time point).
