# Supplementary figures and images for: A Novel Peptide Enhances Therapeutic Efficacy of Liposomal Anti-Cancer Drugs in Mice Models of Human Lung Cancer
Source: PLoS One. 2009 Jan 12;4(1):e4171. doi: 10.1371/journal.pone.0004171 (PMC2614347; doi:10.1371/journal.pone.0004171)

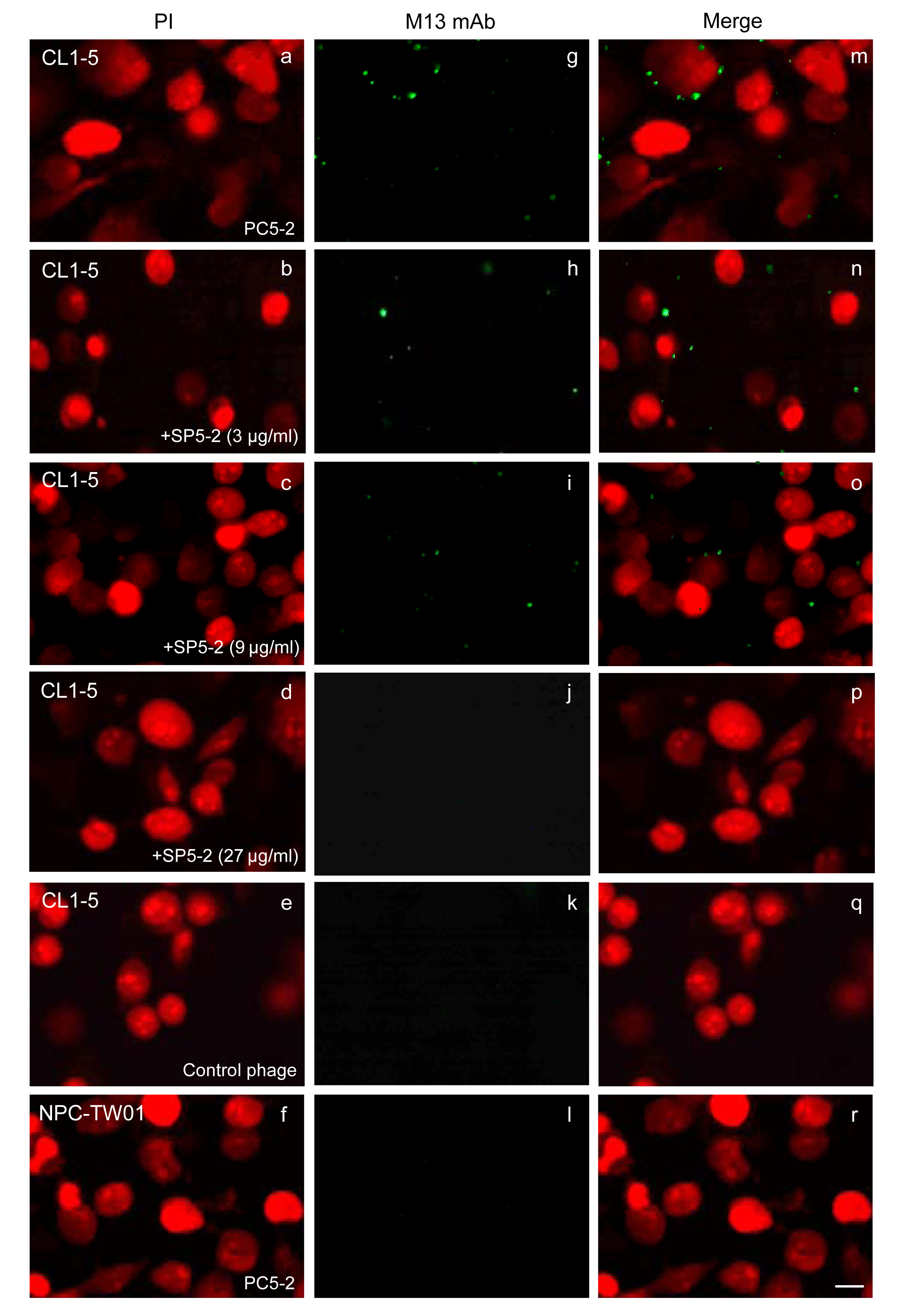

Supplement: Figure S1 — Identification of PC5-2 binding to NSCLC cells. Representative microscopy images of CL1-5 cells stained with propidium iodide (a–f, red), anti-M13 monoclonal antibody (g–l, green), and merge (m–r). The binding of PC5-2 to CL1-5 cells (a, g, m) was inhibited by 3 µg/ml (b, h, n), 9 µg/ml (c, i, o), and 27 µg/ml (d, j, p) of SP5-2 in a dose-dependent manner. The control phage and PC5-2 did not bind to CL1-5 cells (e, k, q) and NPC-TW01 cells, respectively. Scale bar: 10 µm. (3.54 MB TIF) [file pone.0004171.s003.tif]

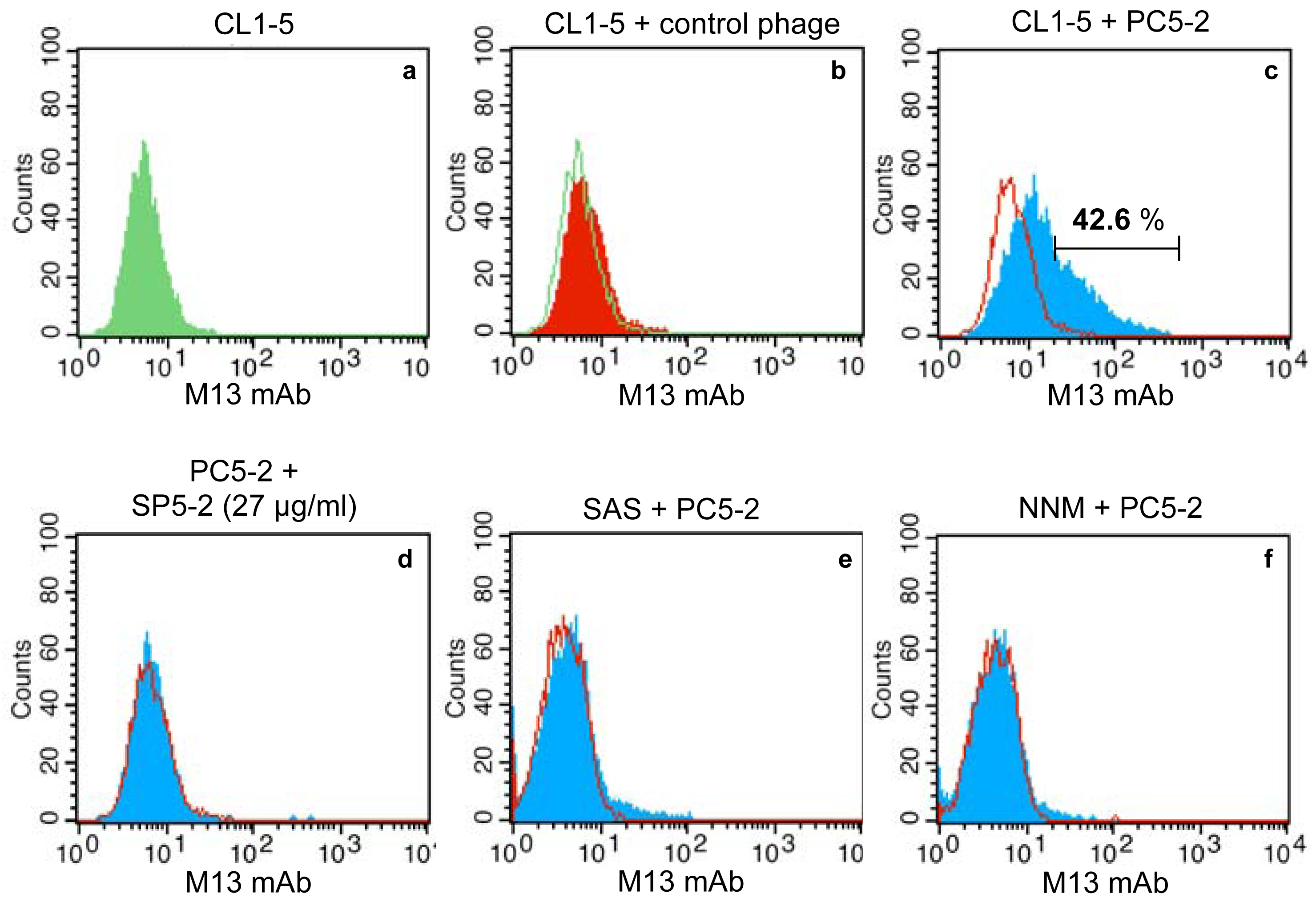

Supplement: Figure S2 — The binding activity of PC5-2 to NSCLC cells was analyzed by flow cytometry. The samples included cells only (a, green), control phage (b, red), and PC5-2 (blue). The percentages indicate the fraction of cells gated for positive binding (c). The binding of PC5-2 to CL1-5 cells was completely inhibited by 27 µg/ml of SP5-2 (d). PC5-2 did not bind to SAS and NNM cells (e, f). (2.42 MB TIF) [file pone.0004171.s004.tif]

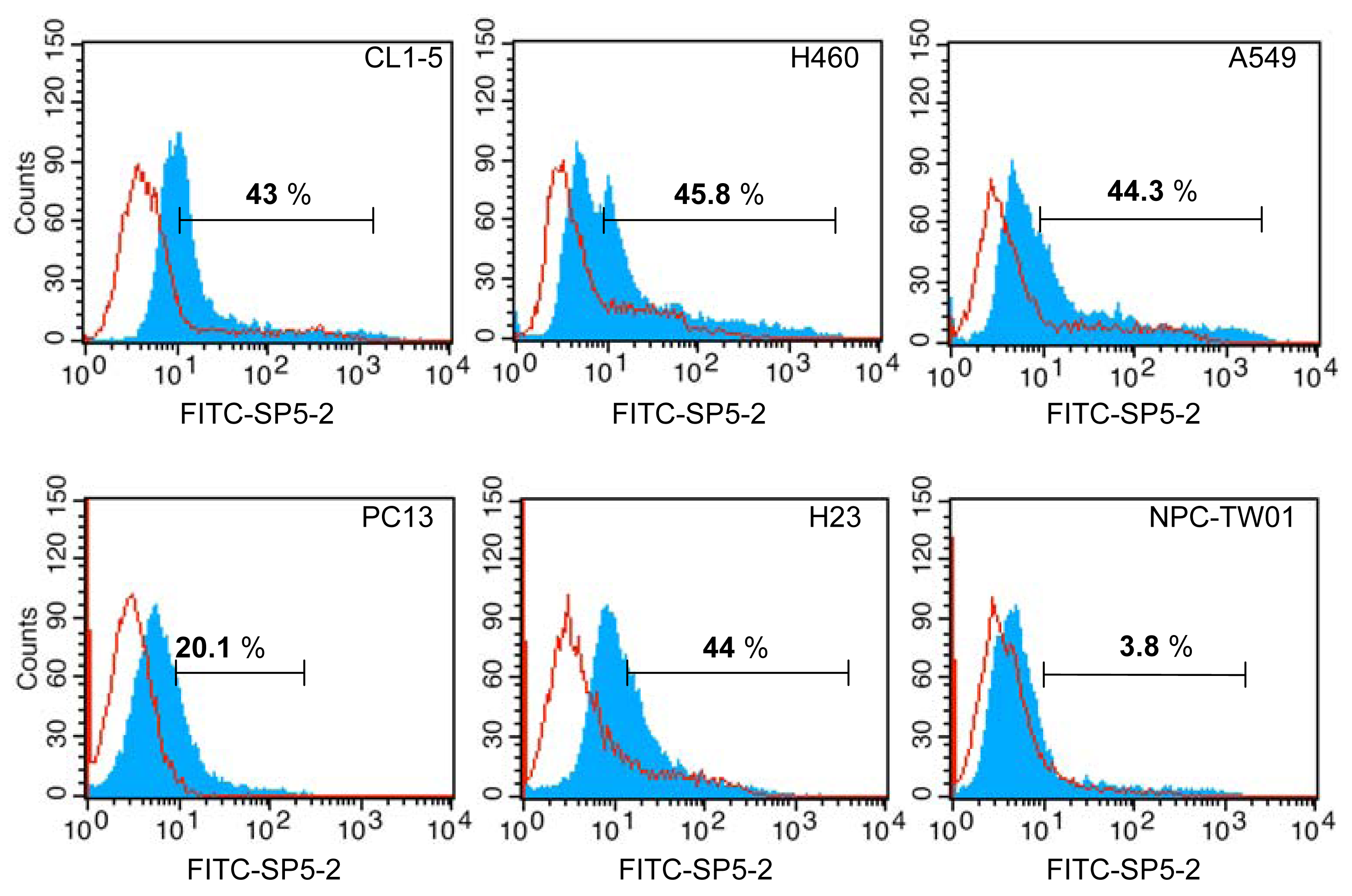

Supplement: Figure S3 — FITC-labeled SP5-2 binding to NSCLC cells was analyzed by flow cytometry. The red line superimposed on all panels represents the control phage. CL1-5, H460, A549, PC13, and H23 panels showed positive binding, and the percentages were 43, 45.8, 44.3, 20.1, and 44, respectively. There was only background level in NPC-TW01 cells. (3.04 MB TIF) [file pone.0004171.s005.tif]

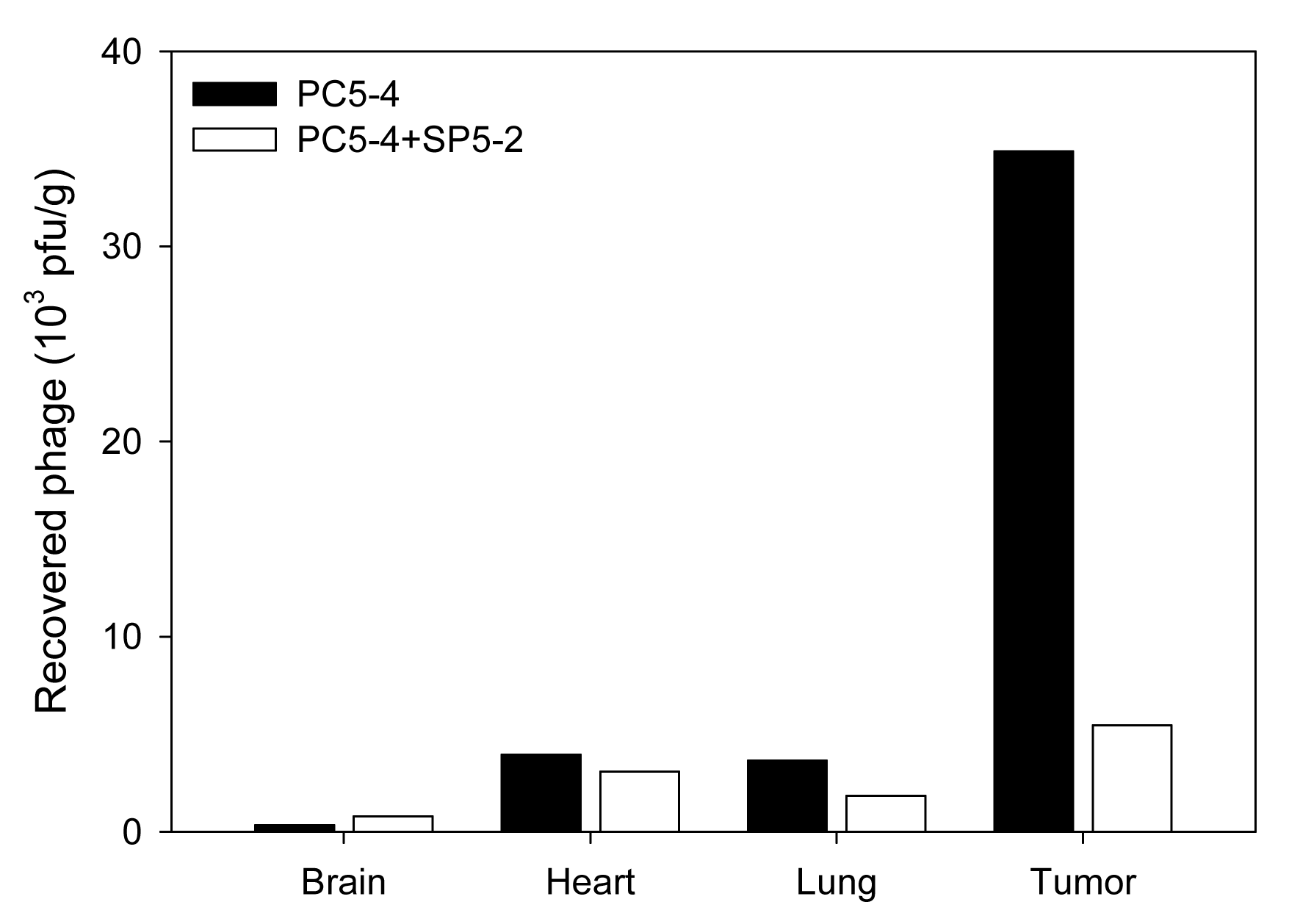

Supplement: Figure S4 — Tumor homing ability of PC5-4 phage. SCID mice bearing NSCLC xenografts were injected i.v. with PC5-4, and phage was recovered after perfusion. Recovery of PC5-4 from the tumor was higher than from control organs. Targeting activity of PC5-4 to tumor tissues was inhibited by SP5-2. (0.18 MB TIF) [file pone.0004171.s006.tif]

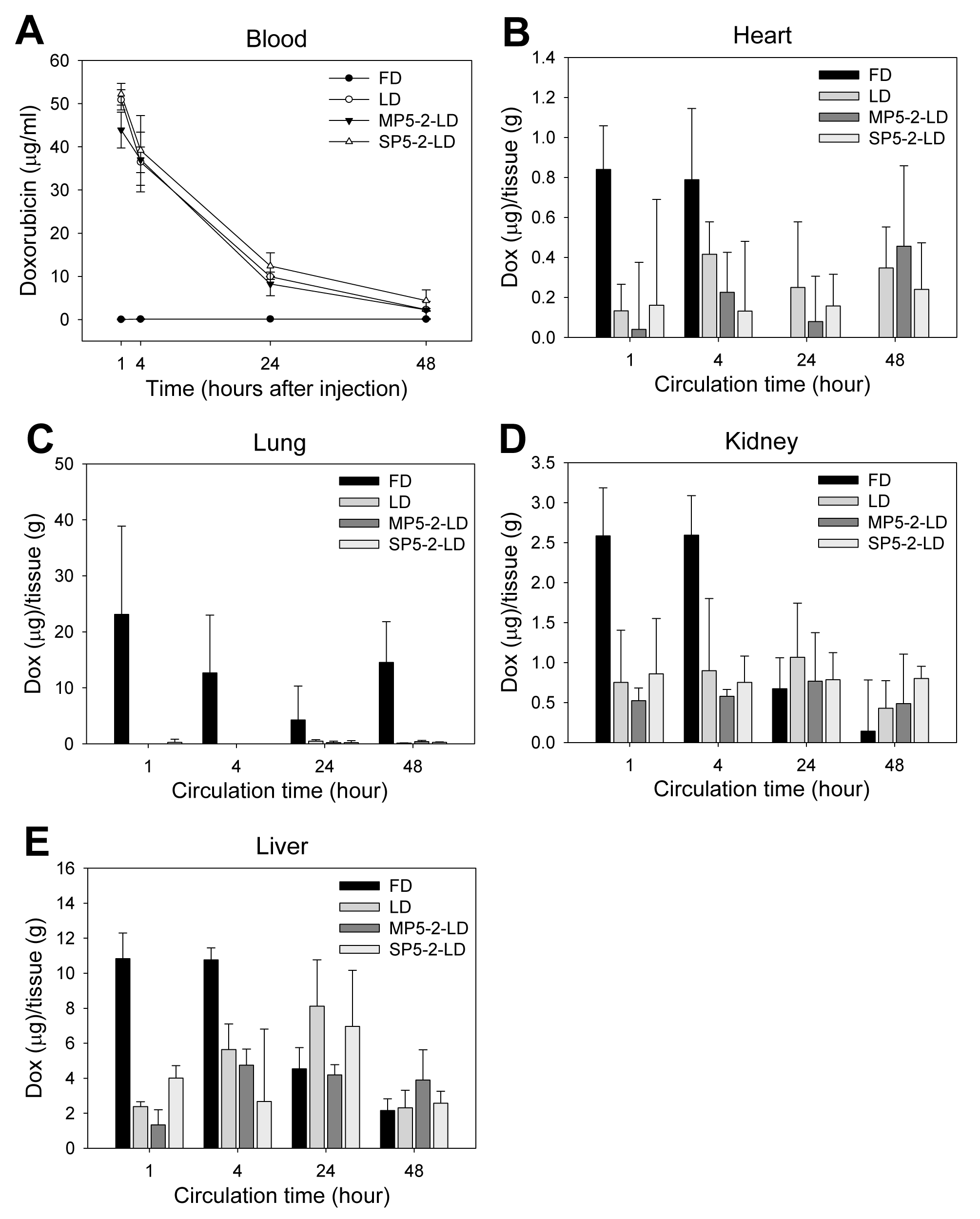

Supplement: Figure S5 — Biodistribution of different formulations of liposomal and free doxorubicin in a NSCLC xenograft mouse model. Mice were i.v. injected with SP5-2-LD, MP5-2-LD, LD, and FD in a single dose of 2 mg/kg. At selected time points (1, 4, 24 and 48 hours) after injection, doxorubicin concentration in blood, and organs were measured (n = 3 at each time point). (0.62 MB TIF) [file pone.0004171.s007.tif]

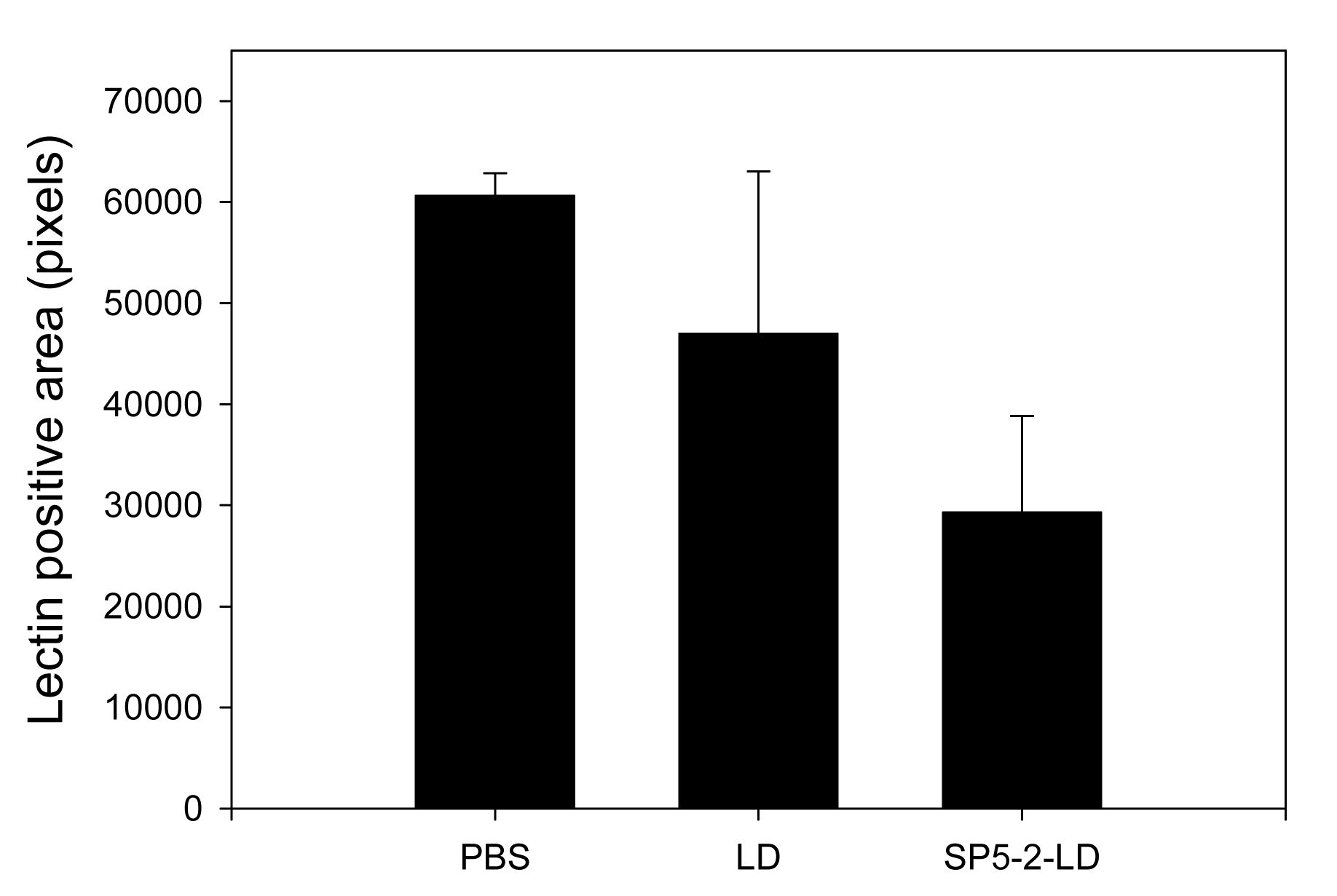

Supplement: Figure S6 — Changes of lectin-reactive vessels in SP5-2-LD-treated tumors. Positive lectin localization was quantified by pixel area count (Metamorph software, MDS, Inc.) under low power magnification. (0.20 MB TIF) [file pone.0004171.s008.tif]
