# Supplementary material for: A Novel Peptide Enhances Therapeutic Efficacy of Liposomal Anti-Cancer Drugs in Mice Models of Human Lung Cancer
Source: PLoS One. 2009 Jan 12;4(1):e4171. doi: 10.1371/journal.pone.0004171 (PMC2614347; doi:10.1371/journal.pone.0004171)
Supplement: Table S1 — Detection of human lung cancer surgical specimens by PC5-2 using immunohistochemistry (0.06 MB DOC) [file pone.0004171.s009.doc]

| **Table S1.** Detection of human lung cancer surgical specimens by PC5-2 using immunohistochemistry | **PC5-2** | **Control phage** |
| --- | --- | --- |
| **HLC 1** | +++ | **-** |
| **HLC 2** | +++ | - |
| **HLC 3** | - | - |
| **HLC 4** | + | - |
| **HLC 5** | +++ | - |
| **HLC 6** | ++ | - |
| **HLC 7** | ++ | - |
| **HLC 8** | ++ | - |
| **HLC 9** | + | - |
| **HLC 10** | - | - |
| **HLC 11** | ++ | - |
| **HLC 12** | ++ | - |
| **HLC 13** | + | - |
| **HLC 14** | - | - |
| **HLC 15** | +++ | - |
| **HLC 16** | - | - |
| **HLC 17** | - | - |
| **HLC 18** | ++ | - |
| **HLC 19** | +++ | - |
| **HLC 20** | +++ | - |
| **HLC 21** | ++ | - |
| **HLC 22** | - | - |
| **HLC 23** | - | - |
| **HLC 24** | ++ | - |
| **HLC 25** | +++ | - |
| **HLC 26** | +++ | - |
| **HLC 27** | + | - |
| **HLC 28** | ++ | - |
| **HLC 29** | - | - |
| **HLC 30** | ++ | - |
| **HLC 31** | ++ | - |
| **HLC 32** | - | - |
| **HLC 33** | ++ | - |
| **HLC 34** | +++ | - |
| **HLC 35** | + | - |
| **HLC 36**  HLC: human lung cancer surgical specimen.  Reaction area, +++: >50%; ++: 50~20%; +: <20%; -: 0%. | ++ | - |
