# Supplementary material for: A Novel Peptide Enhances Therapeutic Efficacy of Liposomal Anti-Cancer Drugs in Mice Models of Human Lung Cancer
Source: PLoS One. 2009 Jan 12;4(1):e4171. doi: 10.1371/journal.pone.0004171 (PMC2614347; doi:10.1371/journal.pone.0004171)
Supplement: TableS2 — Tumor pharmacokinetics of free doxorubicin versus liposomal doxorubicin formulations (0.03 MB DOC) [file pone.0004171.s010.doc]

| **Formulation (2 mg/kg)** | **Free Doxorubicin .** | | **Liposomal Doxorubicin** | | **MP5-2-LD .** | | **SP5-2-LD .** | |
| --- | --- | --- | --- | --- | --- | --- | --- | --- |
|  | **Total** | **Nuclear** | **Total** | **Nuclear** | **Total** | **Nuclear** | **Total** | **Nuclear** |
| AUC_0-48 h_ (μg h/g tumor) | 10.44 | 3.7 | 31.48 | 6.72 | 29.03 | 7.45 | 59.87 | 17.68 |

**Table S2.** Tumor pharmacokinetics of free doxorubicin versus liposomal doxorubicin formulations
